# The endothelial layer formation in the presence of AuNPs/CdSe/TaNPs-loaded PLCL/PVP-based electrospun nanofibers

**DOI:** 10.3389/fmolb.2025.1638442

**Published:** 2025-08-18

**Authors:** Magdalena Lasak, Viraj P. Nirwan, Dorota Kuc-Ciepluch, Ryszard Tomasiuk, Igor Chourpa, Amir Fahmi, Karol Ciepluch

**Affiliations:** ^1^ Division of Medical Biology, Jan Kochanowski University in Kielce, Kielce, Poland; ^2^ Faculty of Technology and Bionics, Rhine-Waal University of Applied Science, Kleve, Germany; ^3^ Department of Basic Medical Sciences, Faculty of Medical Sciences and Health Sciences, Casimir Pulaski University of Radom, Radom, Poland; ^4^ EA 6295 Nanomédicaments et Nanosondes, Faculté de pharmacie, Université de Tours, Tours, France

**Keywords:** nanofibers, gold nanoparticles, quantum dots, tantalum nanoparticles, endothelium

## Abstract

**Background:**

Electrospun nanofibers, which are becoming increasingly popular in biomedicine, can directly or indirectly affect the properties and formation of the edothelial layer. This effect can be both toxic and pro-stimulatory. Therefore, in this study, electrospun nanofibers PLCL/PVP composed of biodegradable and biocompatible L-lactide-block-*ϵ*-caprolactone copolymer (PLCL, 70:30) blended with polyvinylpyrrolidone (PVP), containing *in situ* synthesized PVP different types of nanoparticles - gold (AuNPs), cadmium selenide (CdSe QDs) or tantalum (TaNPs), were investigated. Understanding how different modifications of nanofibers can affect the formation of the endothelial layer is crucial to using them as tools in tissue regeneration.

**Methods:**

electrospun nanofibers with gold (AuNPs), cadmium selenide (CdSe QDs) or tantalum (TaNPs), were synthesized and physico-chemical characteristic were caried out. Cytotoxicity and prostimulatory effect of nanofibers on Primary Human Umbilical Vein Endothelial Cells were tested by microscopic and spectrofluorescence techniques.

**Results:**

The endothelial layer forms to 75% confluence (after 24 h) and reaches 100% after 72 h when no nanofibers are present. A slower formation of the endothelial layer is seen in the presence of PLCL/PVP nanofibers (60%) and (80%) after 72 h. The introduction of various nanoparticles into the nanofibers caused changes in the morphology and rate of endothelial layer formation. In the presence of nanofibers modified with AuNPs after 72 h it reached only 40%. A similar effect was obtained for PLCL/PVP-CdSe QDs. In the case of PLCL/PVP-TaNPs, after 48 h 90% and after 72 h 100%. The tested nanofibers did not show toxic behavior towards the formed HUVEC cell monolayer. All of the tested nanofibers, except PLCL/PVP-TaNPs, induced increased HUVEC cells layer permeability, which resulted in increased translocation of fluorescently labeled dextran from 20% to 50%.

**Conclusion:**

It was estimated that the effect of nanofibers on the formation of the endothelial layer can be direct, where cells contact the nanofibers and thus the growth of the endothelium is hindered. Additionally, the uptake of biological fluid components can have an indirect effect on endothelial cells, their adhesion and growth. Among the tested nanofibers, non-toxic PLCL/PVP-TaNPs seem to be particularly promising due to safety issues and the possibility of using them as effective scaffolds.

## 1 Introduction

Since Nanotechnology has become more popular in many fields of science, especially in biology and medicine, the market is flooded with more and more medical products based on nanomaterials. In 2022 alone, according to the European Observatory for Nanomaterials (EUON), 2,200 nanomaterials-based products were already available on the European market (Study of the EU market for nanomaterials, including substances, uses, volumes and key operators; https://euon.echa.europa.eu/reports). In order to develop effective nanotools for use in anticancer therapies (Shi et al., 2017; van der Meel et al., 2019; Wang et al., 2024), antibacterial (Gold et al., 2018; Saravanan et al., 2023; Skrzyniarz et al., 2023) and diagnostics (Tang et al., 2024; Wang et al., 2024) research is being conducted on nanomaterials and their modifications. Despite numerous reports, knowledge about the toxicity of nanocompounds and their impact on the human body is still limited. Nanomaterials interact with body’s cells and tissues, which can lead to various, often uncontrolled reactions, inducing damage or inflammation of the organism (Hirai et al., 2016; Egbuna et al., 2021; Wu et al., 2021; Kuc-Ciepluch et al., 2022; El-Kady et al., 2023; Märkl et al., 2024). However, it is important, that regardless of the route of administration of nanocompounds, they eventually enter the bloodstream, interacting with blood components, as well as with endothelial cells (ECs) (Setyawati et al., 2013; Ni et al., 2022; Lasak and Ciepluch, 2023a; Huang et al., 2024).

The vascular endothelium, as a single layer of mesenchymal cells lining the inner layer of blood vessels, plays a key role in our body. This highly selective cells monolayer acts as a permeability barrier between blood and tissues, controlling the bidirectional transport of molecules and ions circulating in the blood and protecting tissues from the penetration of dangerous substances (Díaz-Coránguez et al., 2017; Rahimi, 2017; Claesson-Welsh et al., 2021). Endothelial cells also perform an endocrine function, and the mediators secreted by them regulate the vessel tension and processes such as blood clotting, angiogenesis and fibrinolysis. Therefore, the proper functioning of the endothelium is essential for maintaining the homeostasis of the entire organism, and its dysfunction can cause various pathological conditions, including atherosclerosis, stroke, diabetes and neurodegenerative diseases (Wang and He, 2024).

One of the nanomaterials that, due to their purpose, can influence the function and properties of the endothelial layer are nanofibers (NF). These materials have a number of advantages in biomedical applications. Firstly, nanofibers are characterized by a high surface-to-volume ratio and ease of functionalization, which allows for efficient loading of nanofibers matrix with various biologically active substances, such as antibacterial, including metal nanoparticles in wound disinfection and regeneration (Yan et al., 2020; Maliszewska and Czapka, 2022; Reyes-guzmán et al., 2024; Xin et al., 2024; Zhao et al., 2024) and anticancer agents (Bonan et al., 2019), growth factors (Jiang et al., 2018; Asiri et al., 2021), drugs (Cleeton et al., 2019) or vitamins (Cheng et al., 2024), thus broadening the spectrum of their action. Secondly, their high porosity, structure mimicking the external cellular matrix (ECM) and good mechanical strength provide adequate structural support for vascularization, cell adhesion and migration (Abadi et al., 2022; Al-Abduljabbar and Farooq, 2023). Hence, these features make them attractive candidates for wound dressing materials, biosensors for detecting biological compounds, as drug delivery systems or scaffolds in tissue engineering (Dutta et al., 2019; Kulkarni et al., 2022; Nirwan et al., 2023; Lasak et al., 2024).

Polymer nanoscaffolds offer a wide range of both natural and synthetic materials for biomedical applications. Unfortunately, they are not without disadvantages. Natural polymers, such as chitosan or collagen, are biocompatible and biodegradable, but their poor mechanical properties and processability raise concerns. Another problem is the risk of transferring microorganisms together with the natural polymer to the patient’s body. Synthetic polymers, including poly (lactic acid) (PLA) or poly (lactic-co-glycolic acid) (PGA), are characterized by increased mechanical strength, but their low interaction with cells is a problem (Anjum et al., 2022). For this reason, attempts are being made to mix different polymers and modify them (e.g., with nanoparticles) in order to obtain effective scaffolds with the desired properties (Behere et al., 2021).

Until now, it has been reported that nanoparticles (NPs) exhibit cytotoxic and genotoxic effects on endothelial cells, which are mainly associated with inflammatory mechanisms and oxidative stress (Fattahi et al., 2023; Cong et al., 2024). Moreover, nanomaterials can directly induce endothelial leakiness (NanoEL effect, Nanomaterials-Induced Endothelial Leakiness), through interaction with VE-cadherin, which is crucial for maintaining adherens junctions between endothelial cells (Setyawati et al., 2023). However, there is little information on the direct or indirect interaction of nanofibers with ECs, which may be due to the relatively early stage of research on them compared to other nanocompounds. Nevertheless, caused by their great potential in the field of medicine, as well as the increasingly common use of nanomaterials in daily life in commercially available products, it is necessary to determine their effect on endothelial cells.

Herein, electrospun PLCL/PVP nanofibers composed of biodegradable and biocompatible copolymer of L-lactide-block-*ϵ*-caprolactone (PLCL, 70:30) blended with polyvinylpyrrolidone (PVP), containing synthesized *in situ* PVP different types of nanoparticles - gold (AuNPs), cadmium selenide (CdSe QDs) or tantalum (TaNPs), were investigated. Electrospinning is relatively cheap and highly reproducible method of nanofibers fabrication, while *in situ* PVP synthesis of NPs provided controlled size distribution of nanoparticles. Three different types of nanoparticles with various potential for biomedical application were selected in order to provide a wider spectrum of information on their effect on endothelial cells. Gold nanoparticles are known for their antibacterial properties (Li et al., 2014), while CdSe QDs exhibit high toxicity towards cancer cells and unique optical properties (Nirwan et al., 2023). In turn, tantalum compounds, appear as ideal radiosensitizers, increasing the sensitivity of cancer cells to radiotherapy (Ji et al., 2022). In this study, the produced nanomats - PLCL/PVP-AuNPs, PLCL/PVP- CdSe QDs, PLCL/PVP- TaNPs, were physicochemically characterized and their effect on endothelial cells was determined.

## 2 Materials and methods

### 2.1 Polymers and nanoparticles

For the fabrication of nanofibers, poly (L-lactide-co-ε-caprolactone) (PLCL) (av. Mw 200 kg) polymer was bought from Purasorb®, Corbion, Netherlands. Polyvinylpyrrolidone (PVP) (Mw 250 kg), analytical grade chloroform, ethanol, gold precursor, HCl, and Cadmium acetate were purchased from Carl Roth, Karlsruhe, Germany. Sodium borohydride and Ta nanoparticles were purchased from Sigma Aldrich. And selenium powder from Alfa Aesar.

### 2.2 Synthesis of PVP templated nanoparticles

The syntheses of nanoparticles were performed *in situ* using a polymer template strategy. PVP polymer was chosen due to its good solubility in both chloroform and ethanol. Moreover, it has the ability to produce solutions with better electrospinnability. Additionally, PVP was used here as co co-spinning agent to assist the generation of nanofibers using PLCL as the matrix. For the synthesis of PVP-templated AuNPs, 0.01 mM HAuCl_4_· 3 H_2_O was solubilized in ethanol, and 3.6 g of PVP was added once the solution was stable. The resulting solution was kept at 30°C and reduced using a freshly prepared 0.1 mM concentration of NaBH_4_. The mixture was stirred for a couple of hours (pink solution) to ensure complete reduction, and the ethanol was evaporated using an oven, and the particles were resuspended in 10 mL of chloroform (Lasak et al., 2024).

The preparation of Cadmium selenide nanoparticles was performed using the methodology described elsewhere (Nirwan et al., 2023). Briefly, Cd acetate was solubilized in PVP ethanol solution under N2 purge. NaHSe solution prepared simultaneously from Se powder and NaBH4 was added dropwise to it until the solution turned yellowish. The resulting CdSe NPs were re-dispersed in chloroform before being suspended in electrospinning solution.

To prepare a suspension from Ta powder, 9 mg powder was added to 5 mL of chloroform and 200 µL 37% HCl. The suspension was kept under the ultrasonic bath for 2 h to ensure a stable suspension. A black colored suspension was obtained that was used to functionalize the electrospinning solution.

The nanoparticles were analyzed using TEM (Transimsion electron microscopy), and the resulting hybrid nanofibers were analyzed using SEM (scaning electron microscopy).

### 2.3 Fabrication of the nanofibers

The generation of nanofibers using electrospinning from various solutions (coelectrospining) was done at optimized parameters already described elsewhere (Nirwan et al., 2022; 2023; Lasak et al., 2024; Kulkarni et al., 2025). For the fabrication of PLCL/PVP fibers, an electrospinning solution of 0.425g PVP added to 1.7g PLCL dissolved in chloroform was used. The electrospinning solution for AuNPs loaded PLCL/PVP fibers was formed by dissolving 0.425 g PVP in 8 mL AuNPs chloroform suspension and 1.7 g PLCL in 8 mL chloroform. The two solutions were blended before electrospinning. Similarly, CdSe NPs loaded fibers were obtained from a blend of solutions by dissolving 0.425 g PVP in 8 mL CdSe NPs chloroform suspension and 1.7 g PLCL in 8 mL chloroform. Finally, Ta NPs loaded fibers were obtained from the solution by dissolving 0.425 g PVP in 3 mL chloroform, adding to it 5 mL Ta NPs suspension and 1.7 g PLCL in 8 mL chloroform. The optimized parameters used for electrospinning and generation of nanofibers are described in Table 1.

**TABLE 1:** Optimized parameters for the generation of pristine and nanoparticle functionalized hybrid nanofibers.

Sample name	Voltage [kV]	Flow rate [mL hr^-1^]	Temperature [ᵒC]	Humidity [%]
BLANK (PLCL/PVP)	16, −4	0.80	18	80
AuNPs-PLCL/PVP	12, −4	1	16	80
TaNPs-PLCL/PVP	10, −4	1	16	80
CdSe NPs-PLCL/PVP	12, −4	1	16	85

### 2.4 Physicochemical characterization of fabricated nanofibers

Physicochemical characterization of the nanofibers has already been performed and can be found in the following articles (Nirwan et al., 2023; Lasak et al., 2024). The morphologies of the unmodified and modified nanofibers were determined using scanning electron microscopy (SEM, JSM-IT 100 InTouchScope^™^, Freising, Germany) at an accelerating voltage of 15 kV.

The samples were placed on carbon-coated 300 mesh copper grids, left to dry completely, coated with gold spray using a JEOL JFC 110E Fine Coat Ion Sputter, and analyzed by SEM. The hydrodynamic diameter of AuNPs, QDs and TaNPs was measured using dynamic light scattering (DLS) in a photon correlation spectrometer (Anton Paar Ligth sizer 500, Austria). The refraction factor was assumed to 1.33 while detection angles were 15°, 90° and 175°, and the wavelength 658 nm. The data were analyzed using Anton Paar software. PBS was used as a solvent. Additionally, Transmission Electron Microscopy TEM microscopy was also done to confirm the size distribution of nanoparticles.

### 2.5 Preparation and sterilization of nanofibers for biological tests

For biological tests, the UV sterilization method of nanofibers was used, as the most preferred and effective according to the literature data (Evrova et al., 2019a). For this purpose, nanofibers were cut into appropriately diameter disks: 6, 22, 35 mm for 96-, 12- and 6-well plates, respectively, to cover the well surface. Then, the disks were exposed to UV light (λ = 254 nm) for 30 min each side. The effect of UV radiation (0.5 h and 24 h) on the morphology of nanofibers was assessed using a Confocal Raman Microscope (LabRam, Horiba) (exc 691 nm; objx50 lwd; 36 × 5s; bin = 1). All spectra were normalized to the same band intensity at 1,435 cm-1. Offset for clarity.

### 2.6 Effect of nanofibers on endothelial cells

#### 2.6.1 Cell growth and morphology in the presence of nanofibers

*In vitro* tests were performed using the HUVEC cell line (Primary Human Umbilical Vein Endothelial Cells, PromoCell). Cells were cultured in complete Endothelial Cell Growth Medium (Cell Applications, Inc.) at 37ᵒC in a humidified atmosphere and 5% CO2. The culture medium was changed every 2 days. To assess cell growth and morphology, HUVEC cells were seeded in 6-well plates and cultured with different types of nanofibers for 24, 48, and 72 h and observed under an optical microscope (64x) at each time point.

#### 2.6.2 Cytotoxic effect of nanofibers on endothelial cells

The viability of HUVEC cells treated with nanofibers was assessed using the MTS Cell Proliferation Assay Kit (Colorimetric) (Abcam). Briefly, cells were cultured in a 96-well plate until the appropriate cell confluence was reached and then treated with nanofibers for 24 h. After that, the MTS test was used according to the manufacturer’s recommendations. Absorbance was measured at 490 nm.

#### 2.6.3 Stability of endothelial monolayer treated with nanofibers

The integrity of the endothelial monolayer treated with nanofibers was assessed using a transwell system equipped with 0.4 μm pore inserts (Corning). For this purpose, HUVEC cells were seeded into the upper chamber of the transwell inserts and cultured until a compact cell monolayer was achieved. After that, nanofibers were added to the lower compartment and incubated for 24 h at 37°C in a humidified atmosphere containing 5% CO2. Then, the nanofibers were removed and replaced with a solution of fluorescein isothiocyanate–dextran (FITC-Dextran, 40 kDa, Sigma-Aldrich) at a final concentration of 1 mg/mL. The plate was incubated in the dark for 20 min at room temperature. The medium from the upper chamber was transferred to a 96-well plate and fluorescence measurement was performed at Ex/Em = 485 nm/525 nm.

## 3 Results

### 3.1 Physicochemical characterization of fabricated nanofibers

Various compositions as described in the methodology were electrospun using the optimized parameters to generate nanofibers. The use of chloroform as the solvent here was prohibited to obtain an interruption free and high-yielding electrospinning process. Due to the very high vapor pressure of the chloroform, constant clogging of the capillary at the spinneret was observed. This led to interruptions during the process as the capillary had to be cleaned, and the clogged polymer needed constant attention. This problem has been described well, and using a solvent mixture should be able to help minimize this issue and improve the electrospinning process (Maduna and Patnaik, 2024; Xing et al., 2024).

**FIGURE 1:**
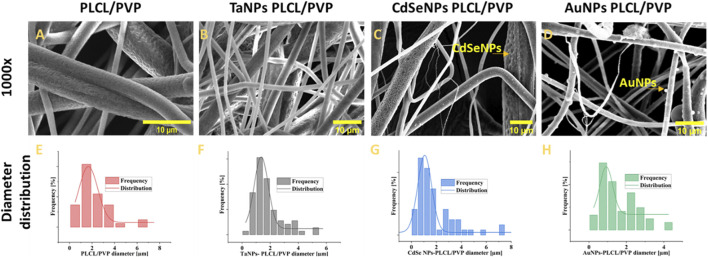
The morphological analysis of fibers under SEM with diameter distribution;, nonfunctionalized **(A,E)** TaNPs functionalized nanofibers, **(B,F)** CdSeNPs functionalized nanofibers **(C,G)**, AuNPs functionalized nanofibers **(D,H)**.

As observed from Figure 1, the morphological analysis of fibers under SEM confirmed the generation of cylindrical fibril structures with dimensions from submicron to a few microns. There was not much change in the diameter distribution of the nanofibers after functionalization with inorganic moieties. The average diameter of pristine nanofibers, as measured using ImageJ, was 2.1 ± 1.4 µm, followed by CdSe functionalized nanofibers at 2.2 ± 2.4 µm. The TaNPs functionalized nanofibers showed an average diameter of 1.82 ± 1 μm and 1.5 ± 0.9 µm was the average diameter measure for AuNPs functionalized nanofibers. The surface of the fibers showed textured topology with variation in depth among various types of fibers. Here, the use of high humidity while performing electrospinning is attributed to the presence of these structures (Xue et al., 2017; Liu et al., 2022). The micro- and macroporous structures observed on the surface of the nanofibers are highly beneficial in the case of biological applications (Leal et al., 2024). These structures provide roughness to the otherwise smooth nanofiber surface, which can improve cell adhesion in the case of tissue regeneration scaffolds. Moreover, these porous structures can act as drug loading sites for the nanofibers, enhancing their loading capacity (Nirwan et al., 2023). Furthermore, via SEM, it was possible to observe the CdSe NPs and AuNPs on the surface of the nanofibers. The surface of TaNPs nanofibers did not show nanoparticles at magnification. The size of the nanoparticles and their morphology were measured using the Dynamic light scattering method and TEM microscopy (S7-S9).

**FIGURE 2:**
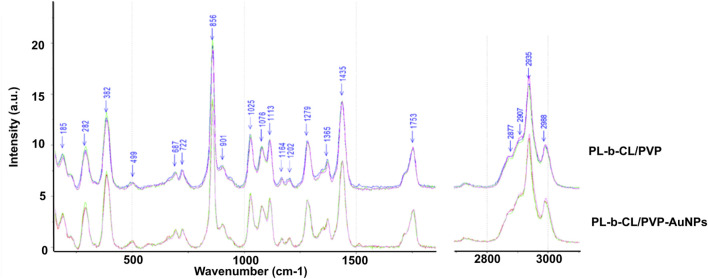
The effect of UV radiation on the morphology of nanofibers after 0.5 h and 24 h of treatment.

For biological tests, all nanofibers were previously subjected to a UV lamp sterilization procedure. Taking into account the fact that the selected sterilization method may affect the properties of the produced nanoscaffolds, their stability after the sterilization process was assessed. Figure 2 shows the effect of UV radiation on the morphology of nanofibers after 0.5 h and 24 h of treatment. As an example, the results for PLCL/PVP and PLCL/PVP - AuNPs nanofibers are presented. As observed, even 24 h exposure of nanofibers to UV light did not cause significant changes in their structure. As expected, a similar result was also observed for 30-min UV treatment of nanofibers.

### 3.2 Effect of nanofibers on endothelial layer formation

**FIGURE 3:**
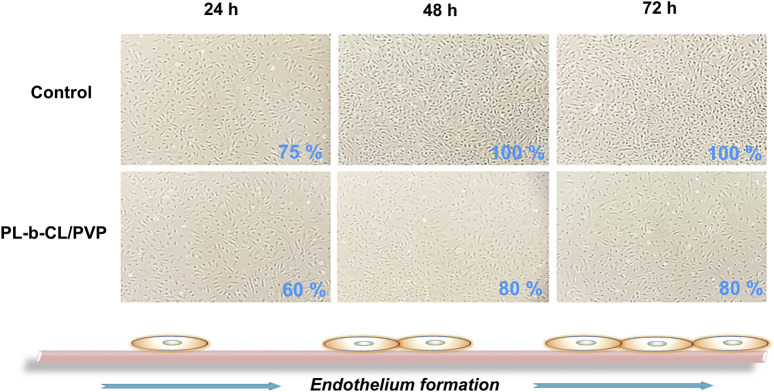
The formation of the endothelial layer in the presence of PLCL/PVP nanofibers compare to cells incubated without nanofibers, after 24, 48 and 72 h.

**FIGURE 4:**
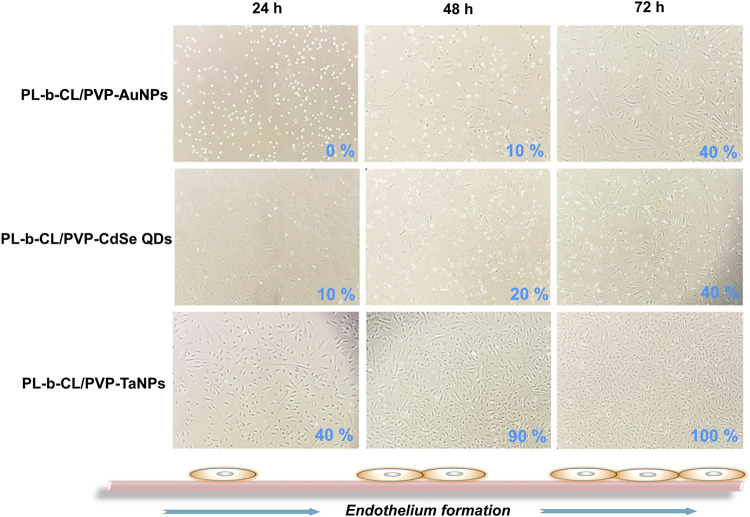
The formation of the endothelial layer in the presence of AuNPs functionalized nanofibers, CdSeNPs functionalized nanofibers and TaNPs functionalized nanofibers after 24, 48 and 72 h.

The growth and morphology of cells in subsequent days of incubation with different types of nanofibers are shown in Figure 3. The endothelial layer forms to 75% confluence (after 24 h) and reaches 100% after 72 h when no nanofibers are present. A slightly slower formation of the endothelial layer is seen in the presence of PLCL/PVP nanofibers. After 24 h, the plate overgrowth is around 60% and after 72 h it reaches only 80%. The introduction of various nanoparticles into the nanofibers caused changes in the morphology and rate of endothelial layer formation (Figure 4). In the presence of nanofibers modified with AuNPs, the cells had problems with attachment to the substrate and did not show the typical morphology of endothelial cells. After 48 h, the plate overgrowth was only 10% and after 72 h it reached only 40%. A similar effect was obtained for PLCL/PVP-CdSe QDs nanofibers. In this case, after 24 h, the plate overgrowth was 10% and after 72 h 40%. In the case of PLCL/PVP-TaNPs, after 24 h the confluence was 40%, after 48 h 90% and after 72 h 100%.

### 3.3 Cytotoxicity of nanofibers on endothelial layer

**FIGURE 5:**
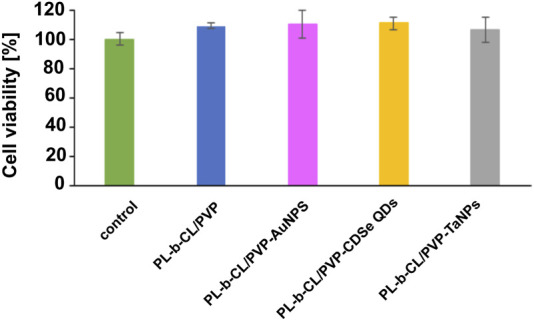
Cell viability of endothelial cells after 24 incubation with different nanofibers measured by MTS Proliferation Assay.

The cytotoxic properties of nanofibers on endothelial cells were studied using the MTS Proliferation Assay. As shown in Figure 5, the tested nanofibers did not show toxic behavior towards the formed HUVEC cell monolayer after 24 h of incubation with different nanofibers, which may suggest their safety in medical applications.

### 3.4 Stability of endothelial layer in presence of nanofibers

**FIGURE 6:**
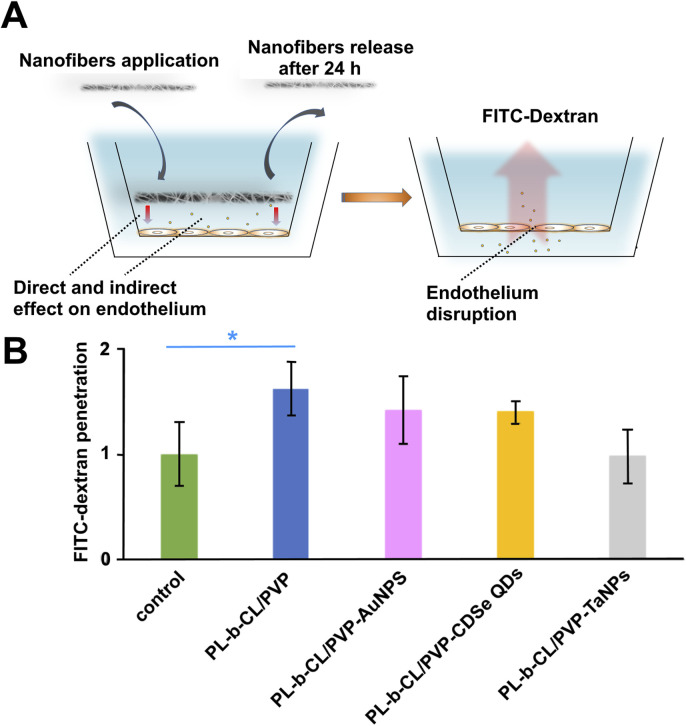
The effect of fabricated nanofibers on the endothelial monolayer. The scheme of experiment **(A)**, HUVEC cell layer permeability caused by increased translocation of fluorescently labeled dextran (FITC-Dextran) **(B)**.

The next step, the effect of fabricated nanofibers on the endothelial monolayer integrity was evaluated. All of the tested nanofibers, except PLCL/PVP-TaNPs, induced increased HUVEC cells layer permeability, which resulted in increased translocation of fluorescently labeled dextran (FITC-Dextran) from 20% to 50% (Figure 6), which confirms the leakiness effect dependent on the type of nanoparticles used. The most significant permeabilization effect is observed in presence of PLCL/PVP alone (up to 50%). The lowest or even no effect was visible for PLCL/PVP modified with TaNPs.

## 4 Discussion

Nanofibers are gaining increasing importance in biomedical applications, including drug delivery, antibacterial and anticancer therapies or effective tissue engineering scaffolds (Chen et al., 2023), which makes their interaction with endothelial cells inevitable (Jiang et al., 2018; Chen et al., 2019; Song et al., 2024). This influence can be both direct, in contact with the endothelial layer, or in the case of new vessel formation, and indirect, when nanofiber components can interact with the endothelial layer or have a real impact on the composition of biological fluids necessary for endothelial formation. Therefore, in this study, we investigated the effect of electrospun PLCL/PVP nanofibers modified with different nanoparticles (gold, cadmium selenide or tantalum) with different potential applications in biomedicine on endothelial cells HUVEC.

For biological tests, all nanofibers were previously subjected to a UV lamp sterilization procedure. Maintaining the correct morphology and structure in potential medical applications is crucial. Available studies show a strong correlation between the type of polymer used and the stability of nanofibers exposed to UV light (Elashnikov et al., 2019; Evrova et al., 2019b; Tort et al., 2020). Considering the fact that the selected sterilization method can affect the properties of the produced nanofibers, their stability was assessed after the sterilization process. Figure 2 shows the effect of UV radiation on the morphology of nanofibers after 0.5 h and 24 h of treatment. As an example, the results for PLCL/PVP and PLCL/PVP - AuNPs nanofibers are presented. As observed, even 24-h exposure of nanofibers to UV light did not cause significant changes in their structure. As expected, a similar result was also observed after 30-min UV treatment of nanofibers.

As mentioned earlier, UV sterilization is crucial and only such nanofibers can be used in biomedicine. The growth and morphology of cells in the subsequent days of incubation with different types of nanofibers after UV sterilization are shown in Figures 3, 4. Cells exposed to PLCL/PVP and PLCL/PVP-TaNPs nanomats maintained normal morphology and growth comparable to the control group, in contrast to HUVEC cells exposed to CdSe QDs nanofibers. The limited growth of endothelial cells exposed to PLCL/PVP-CdSe QDs nanofibers observed in this study may be due to the toxic effects of cadmium compounds (Hu et al., 2021; Rodríguez-Fragoso et al., 2024). However, the application of PVP coating as well as the immobilization of nanoparticles in the nanofiber matrix reduced the toxic effects of CdSe quantum dots while maintaining, albeit limited, cell growth.

In turn, tantalum-based materials are very common in bone tissue engineering, as scaffolds supporting cell growth, with very good mechanical properties and biocompatibility (Zhao et al., 2021; Nan et al., 2022). Nan et al. showed that electrospun scaffolds made of poly-ε-caprolactone (PCL) with tantalum nanoparticles (TaNPs) and magnesium nanooxide (MgO), due to their high osteogenic activity, can be ideal candidates in the treatment of bone defects, which was confirmed by *in vitro* and *in vivo* tests. Moreover, the developed scaffolds stimulated the proliferation of endothelial progenitor cells (EPCs) and angiogenesis, indicating their possible application in vascular tissue engineering (Nan et al., 2022). Fortunately, our studies also showed that cells treated with PLCL/PVP-AuNPs nanofibers needed a longer adaptation time to the substrate than cells treated with other nanofibers. Cell adhesion occurred only after 48 h, and after 72 h, their significant growth with typical morphology was observed. Mohamed et al., studying gold nanoparticles coated with polyvinylpyrrolidone, observed that they inhibited the viability and proliferation of bovine aortic endothelial cells (BAECs) and the phosphorylation of the ERK1/2 pathway, which is important in the regulation of endothelial homeostasis (Mohamed et al., 2017). Among other reasons for reduced HUVEC cell viability in response to gold nanoparticles, disruption of important pathways for endothelial function, such as VEGF-A/VEGFR, has also been indicated (Darweesh et al., 2019). In summary, the incorporation of nanoparticles with different properties into nanofibers affects the biological activity of the entire scaffold and thus induces a different behavior of endothelial cells in the presence of the applied nanofibers. Therefore, different rates of cell proliferation and endothelial layer formation may result from both cell contact with nanofibers, interception of medium components (biological fluid), which may affect the condition of cells, and possible release of nanoparticles from nanofibers. The cytotoxic properties of nanofibers on endothelial cells were studied using the MTS Proliferation Assay. As shown in Figure 5, the tested nanofibers did not show toxic behavior towards the formed HUVEC cell monolayer after 24 h of incubation with different nanofibers, which may suggest their safety in medical applications. These results are consistent with our previous studies, which confirmed the lack of cytotoxic effect of PLCL/PVP and PLCL/PVP-AuNPs nanofibers on human fibroblast cell line (VH10) (Lasak et al., 2024). However, PLCL/PVP-CdSe QDs nanoscaffolds, which showed high cytotoxicity against human lung carcinoma A549 cells in our prior tests (Nirwan et al., 2023), in this study, were not toxic to endothelial cells. Several studies have been conducted to confirm that the toxicity of CdSeQDs is dose, size and surface chemistry dependent, and the mechanism of their toxicity is based on the generation of ROS (reactive oxygen species) and the induction of oxidative stress in cells. Nevertheless, due to their great potential in cancer therapies or bioimaging, various strategies are being undertaken to reduce their toxicity (Kumari et al., 2018; Lin and Chen, 2023; Rodríguez-Fragoso et al., 2024). This was studied by Tang et al. who showed that modification of CdSe/ZnS core–shell QDs with polyethylene glycol (PEG) significantly reduces their *in vitro* and *in vivo* toxicity compared to QDs coated with the cationic polymer polydiallyldimethylammonium chloride (PDDA) (Tang et al., 2013). Our proposed method of *in situ* PVP nanoparticles synthesis, as well as immobilization in a nanofiber matrix, also works in a similar way, reducing the cytotoxic effect on endothelial cells. However, the decreased sensitivity of HUVEC to the toxic effects of PLCL/PVP-CdSe QDs scaffolds observed in this study, compared to other cell types, may also result from the increased resistance of endothelial cells to oxidative stress with subsequent subcultures. It has been known, the decreased sensitivity of HUVEC to oxidative damage correlates with a decrease in cellular iron content, which mediates oxidative stress (Qian et al., 2010). Freshly harvested human umbilical vein endothelial cells contain a significant amount of iron, but the level decreases with subsequent passages, causing increased cell resistance to oxidative damage (Sullivan, 2005).

Nanofibers may be ideal scaffolds for blood vessel regeneration and repair, as developed by Kong et al. biomimetic gelatin (Gt)/polycaprolactone (PCL) composite nanofibers containing chondroitin sulfate (CS) (Kong et al., 2021). However, exposure of cells to nanoparticles incorporated into nanomats can lead to destabilization of cell-cell interactions and increased monolayer permeability. It is also possible that nanoparticles will be released from the nanomats and interact directly or indirectly with blood vessels, as well as the nanofibers topography itself may influence on the interaction with endothelial cells (Cao, 2018; Liu et al., 2018; Lasak and Ciepluch, 2023b). Therefore, in the next step, we investigated the effect of fabricated nanofibers on the endothelial monolayer integrity. All of the tested nanofibers, except PLCL/PVP-TaNPs, induced increased HUVEC cells layer permeability, which resulted in increased translocation of fluorescently labeled dextran (FITC-Dextran) (Figure 6), which confirms the leakiness effect dependent on the type of nanoparticles used. Liu et al. reported a size-dependent effect of gold nanoparticles on vascular endothelial leakiness. They confirmed that 20 nm gold nanoparticles had no significant effect on cell viability, but induced more than 50% increase in HUVEC cell layer permeability due to changes in the cytoskeletal structure, including actin rearrangement, stress fiber formation, and actomyosin contraction (Liu et al., 2017). These results were consistent also in their subsequent work, where they evaluated titanium dioxide, silicon dioxide and polystyrene (NP) nanoparticles with sizes in the range of 20–30 nm, thus confirming the permeabilization effect dependent on the nanoparticle size (Liu et al., 2018).

## 5 Conclusion

**FIGURE 7:**
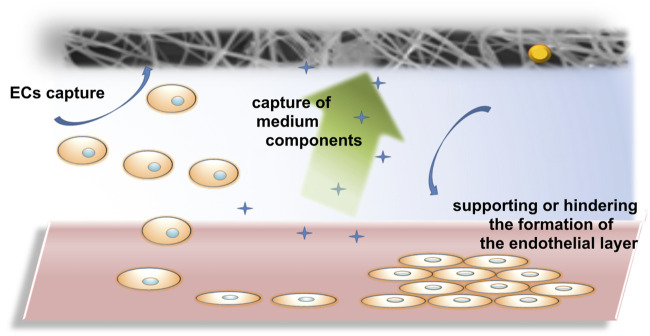
Proposed mechanism of Endothelial layer formation in presence of electrospun nanofibers.

The biological tests performed allowed us to assess the effect of electrospun PLCL/PVP nanofibers modified with nanoparticles on HUVEC cells, which sheds new light on the toxicity of the nanomaterials used and their interactions with the endothelium, which can be used to develop new solutions in nanomedicine. The incorporation of nanoparticles into the nanofiber matrix can affect many biological aspects, including cell growth, proliferation or adhesion, as well as mechanical properties of the scaffolds. It was estimated that the effect of nanofibers on the formation of the endothelial layer can be direct, where cells contact the nanofibers and thus the growth of the endothelium is hindered. Additionally, the uptake of biological fluid by nanofibers can have an indirect effect on endothelial cells, their adhesion and growth (Figure 7). Among the tested nanofibers, non-toxic PLCL/PVP-TaNPs nanomats seem to be particularly promising due to safety issues and the possibility of using them as effective scaffolds. Moreover, the lack of influence of the remaining nanomats on the viability of endothelial cells, together with the possibility of causing leakage of the cell monolayer, can be used in the delivery of drugs to target sites, to which access is difficult. Therefore, our presented results are not only a source of information on the cytocompatibility of the developed nanofibers, but also demonstrate their great potential as nanobiomedical materials.

## Data Availability

The raw data supporting the conclusions of this article will be made available by the authors, without undue reservation.
