# Supplementary material for: The endothelial layer formation in the presence of AuNPs/CdSe/TaNPs-loaded PLCL/PVP-based electrospun nanofibers
Source: Front Mol Biosci. 2025 Aug 18;12:1638442. doi: 10.3389/fmolb.2025.1638442 (PMC12399384; doi:10.3389/fmolb.2025.1638442)
Supplement: Supplementary file 1 [file Supplementaryfile1.docx]

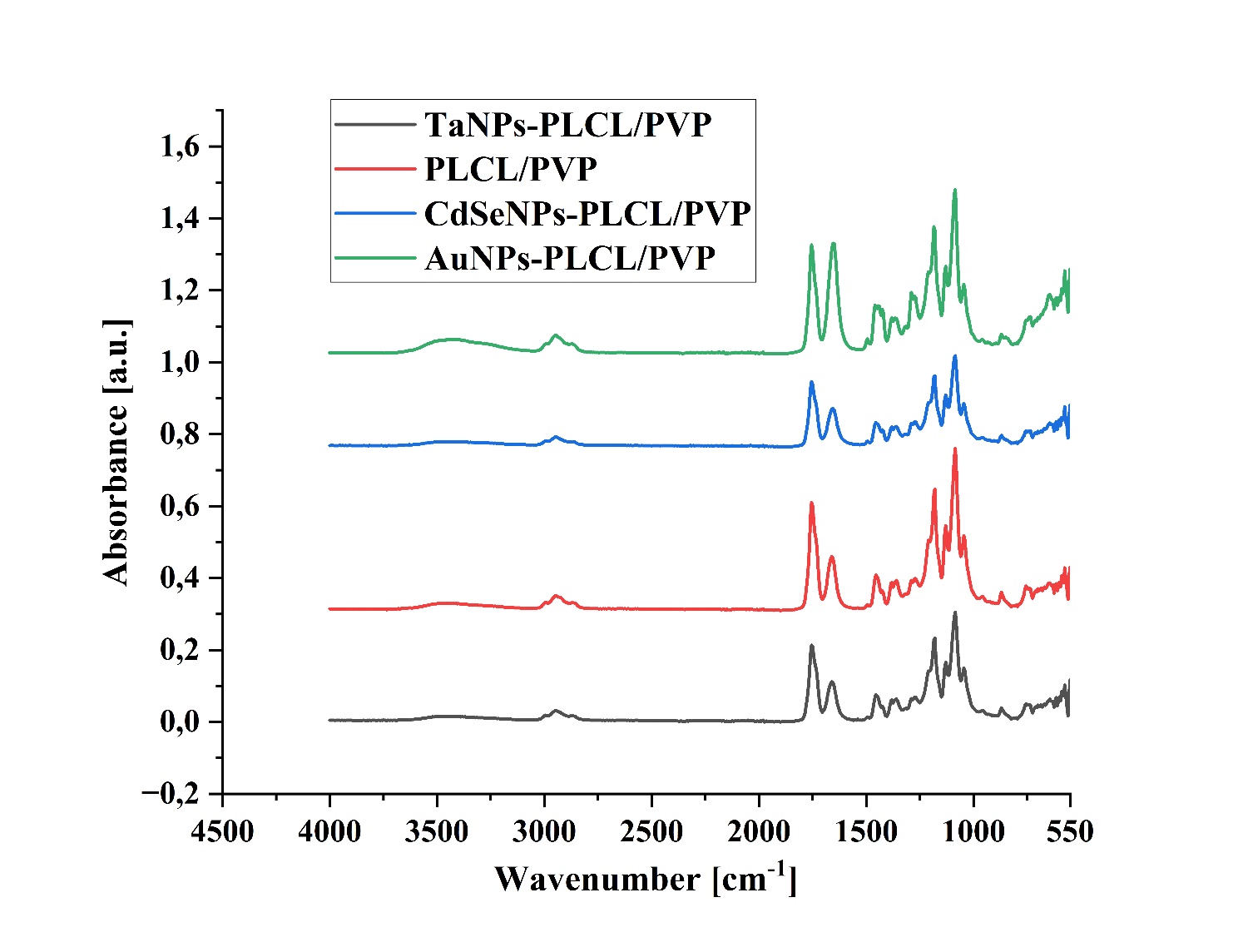


Figure S1 ATR-FTIR analysis of TaNPs functionalized nanofibers and their comparison with AuNPs, CdSeNPs functionalized nanofibers, and pristine nanofibers.


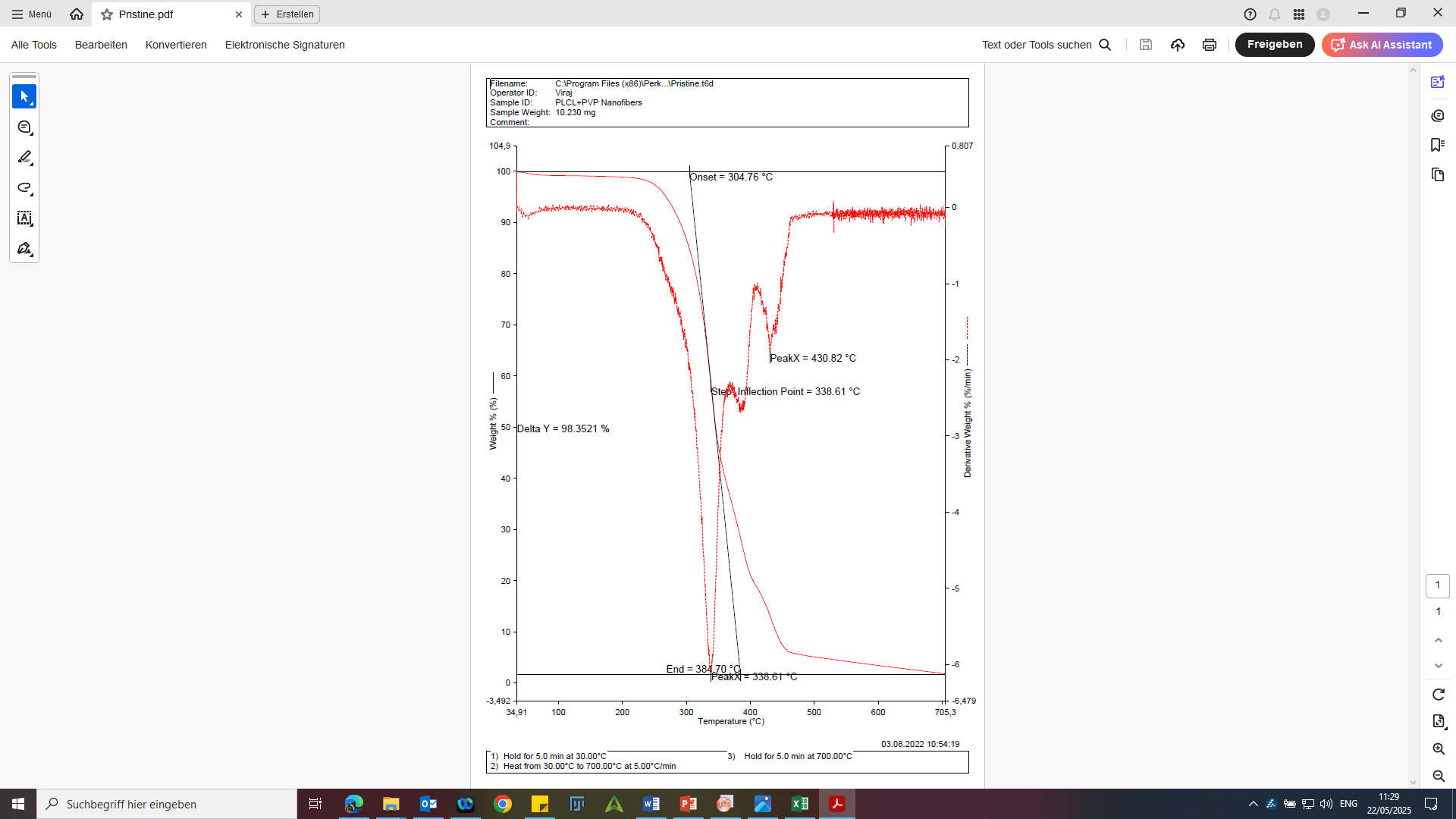


Figure S2 TGA of pristine PLCL/PVP Nanofibers.


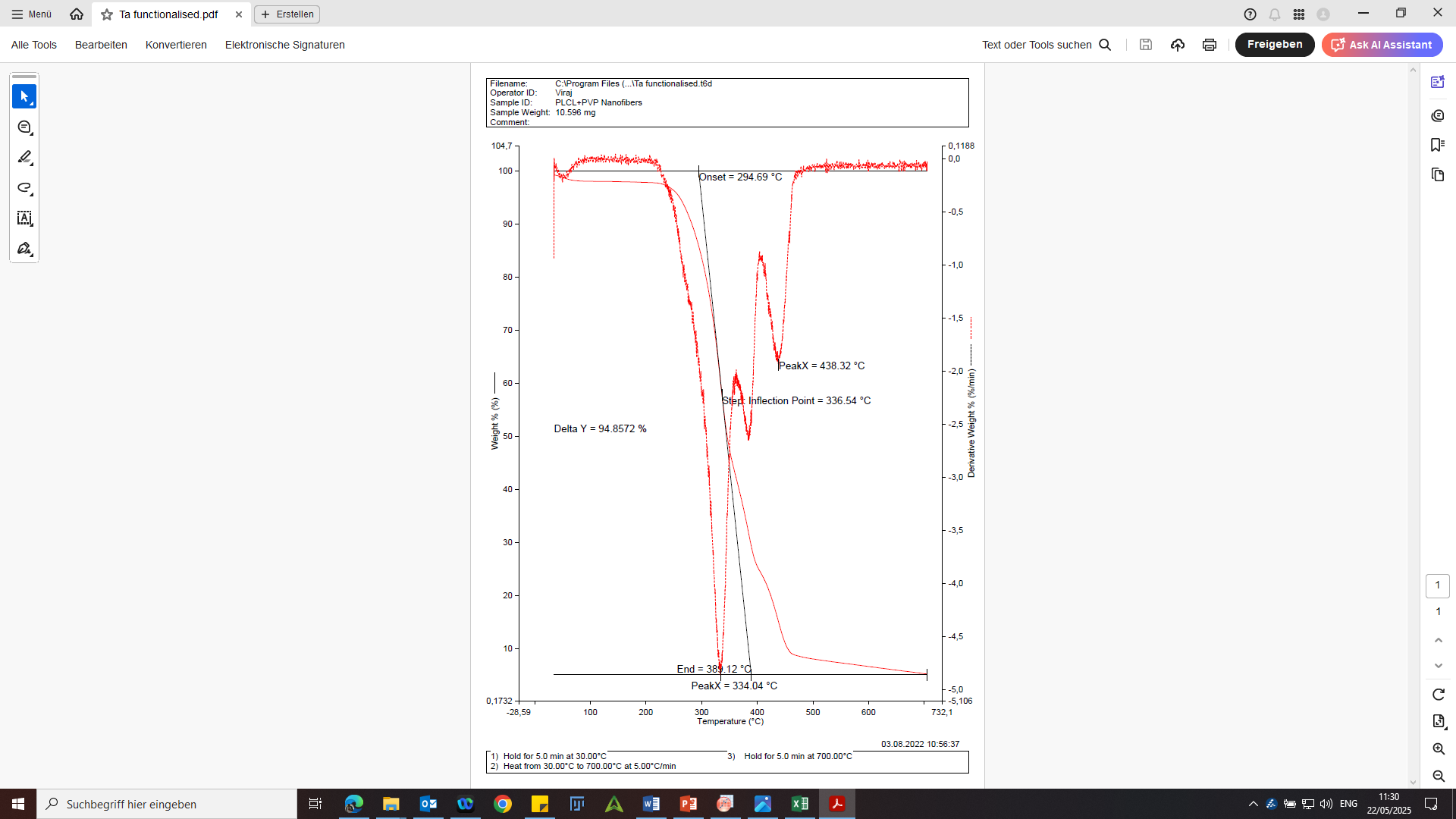


Figure S3 TGA of TaNPs functionalized Nanofibers.


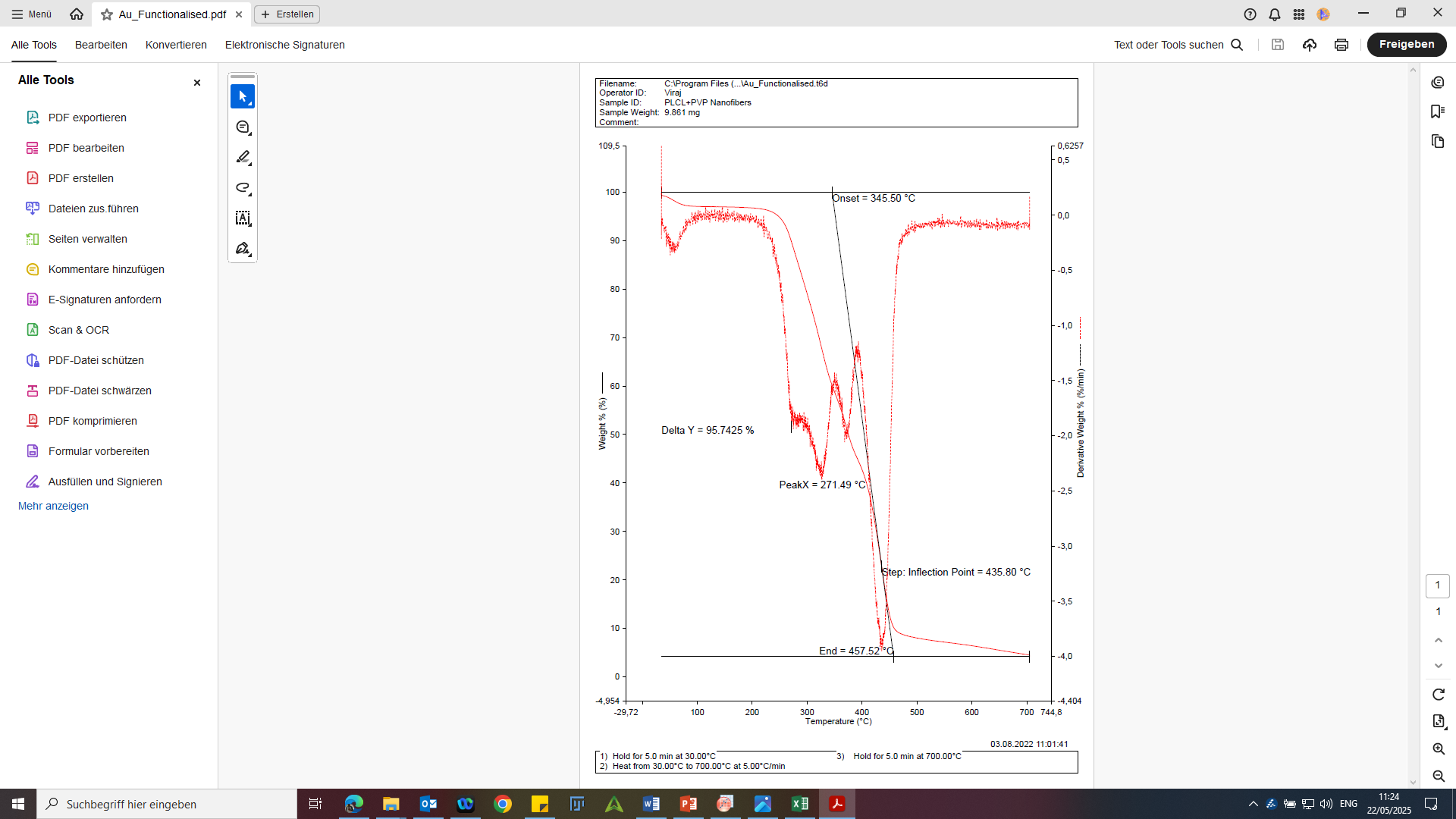


Figure S4 TGA of AuNPs functionalized Nanofibers.


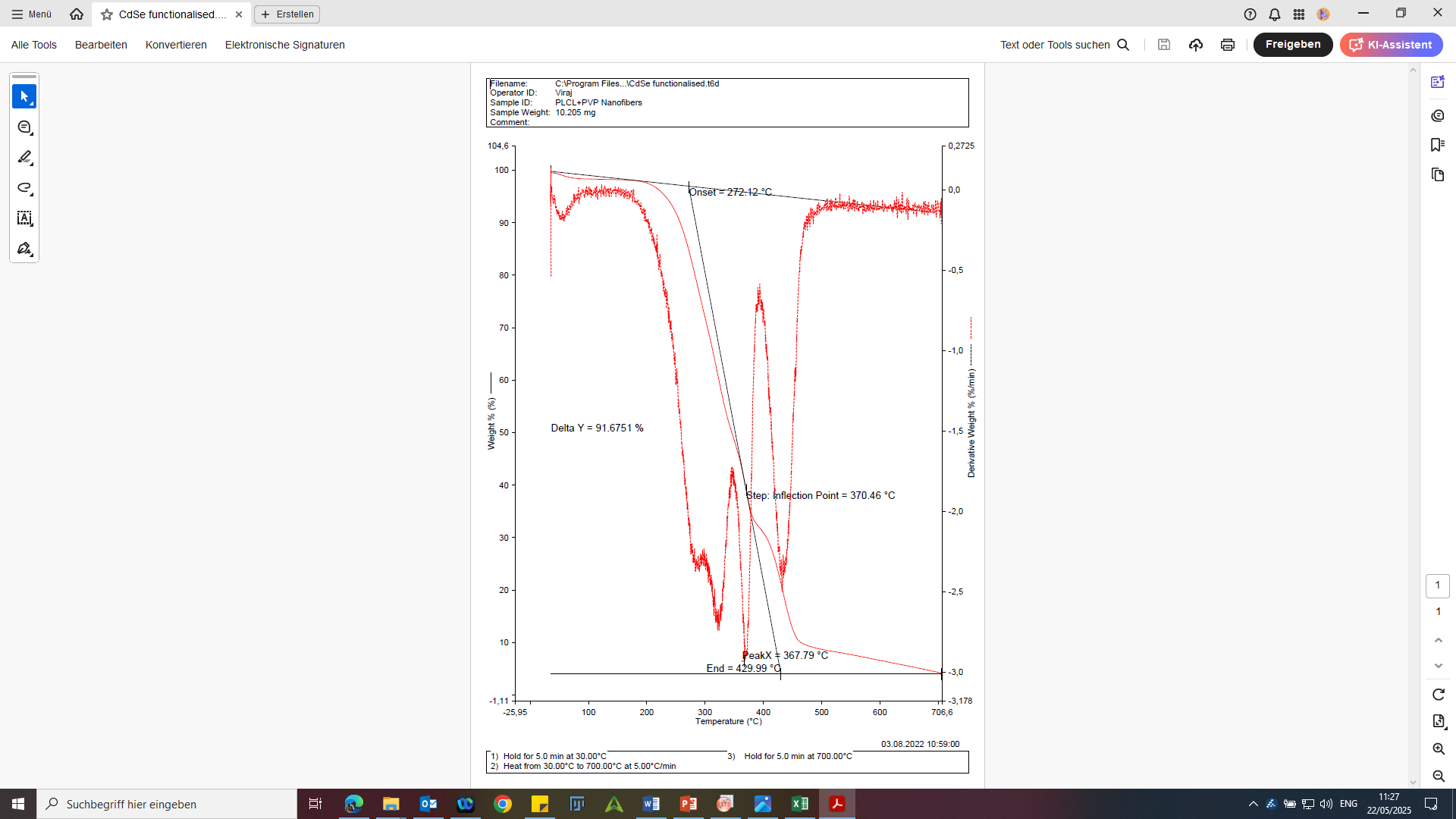


Figure S5 TGA of CdSeNPs functionalized Nanofibers.


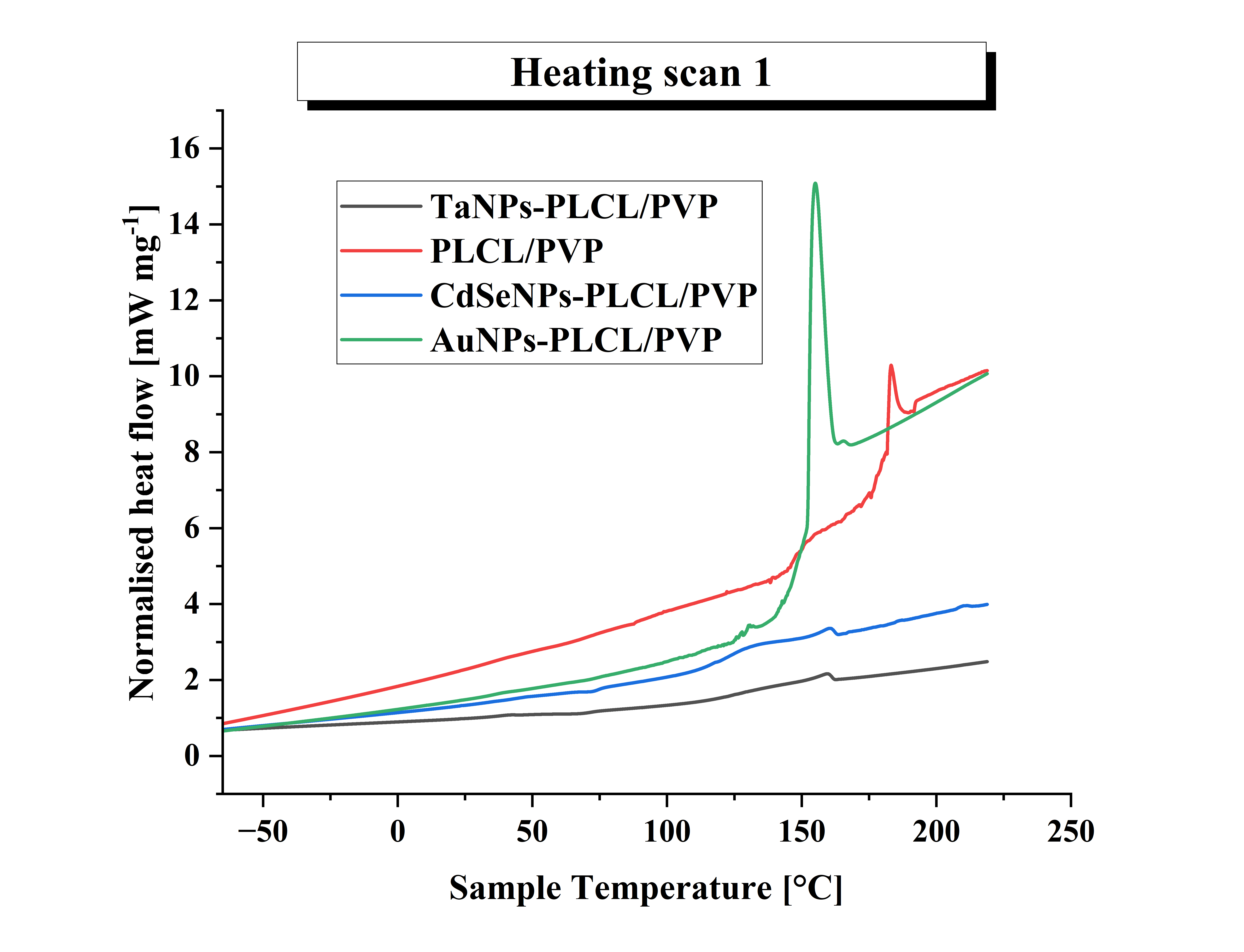


Figure S6 Differential calorimetry of TaNPs functionalized nanofibers and their comparison with counterparts- AuNPs, CdSeNPs functionalized nanofibers, and pristine nanofibers.


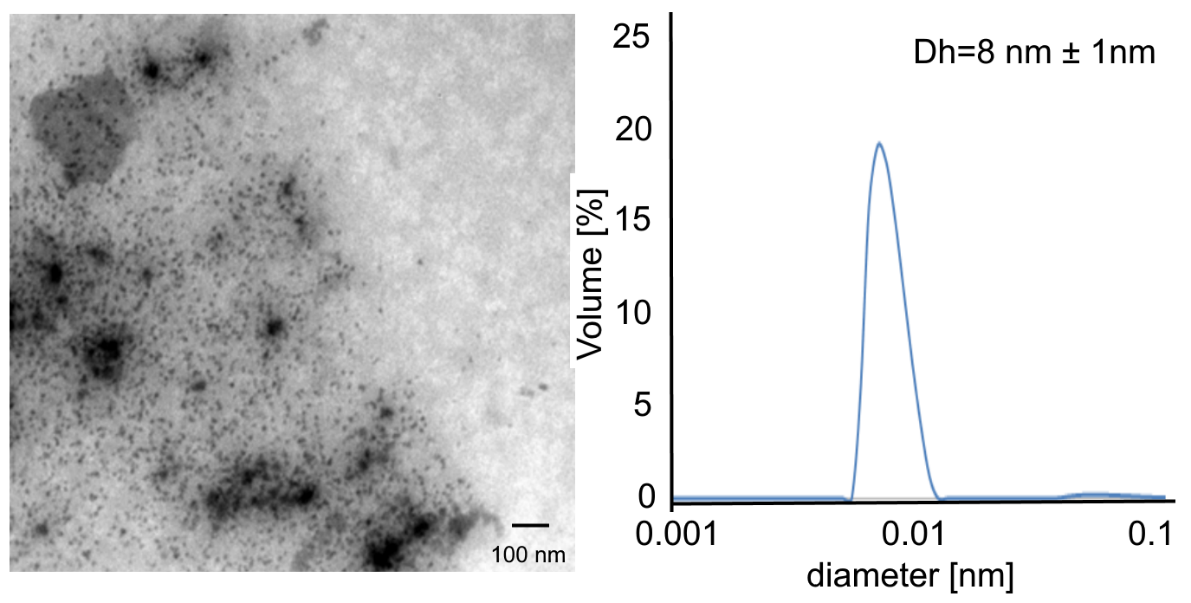


**FigureS7. TEM micrograph and DLS size distribution of AuNPs used for nanomat. (doi.org.10.1002/mabi.202300513)**


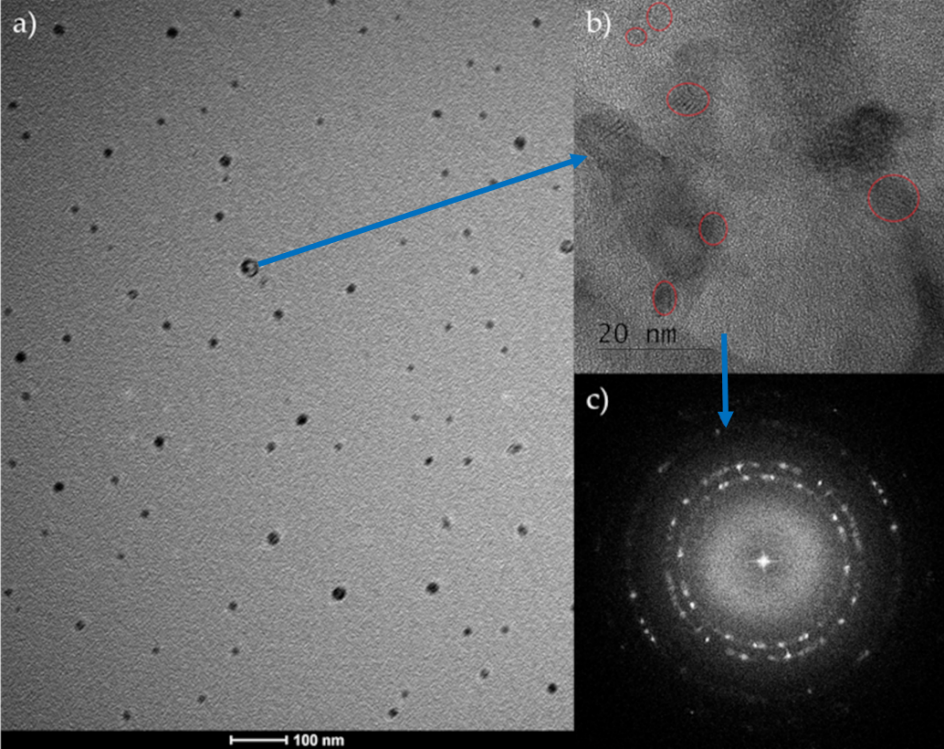


**Figure S8. TEM micrographs of CdSe QDs, a) TEM Overview, b) HR-TEM, c) diffraction pattern of the HRTEM micrograph. (doi.org.10.3390/nano13040630)**


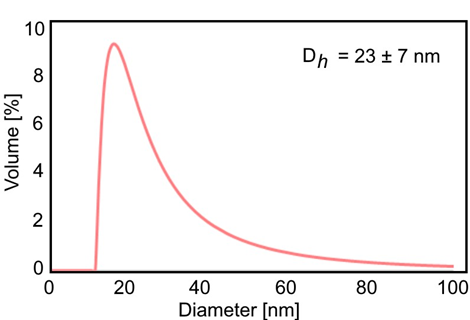


**Figure S9. DLS graph of PVP stabilized CdSe QDs, demonstrating homogeneity with PDI of 0.27. (doi.org.10.3390/nano13040630)**


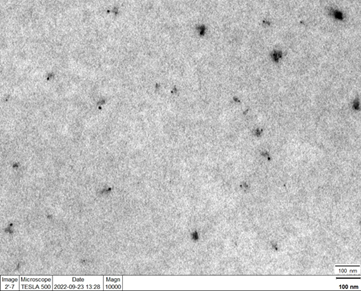


**Figure S10. TEM micrographs of TaNPs**


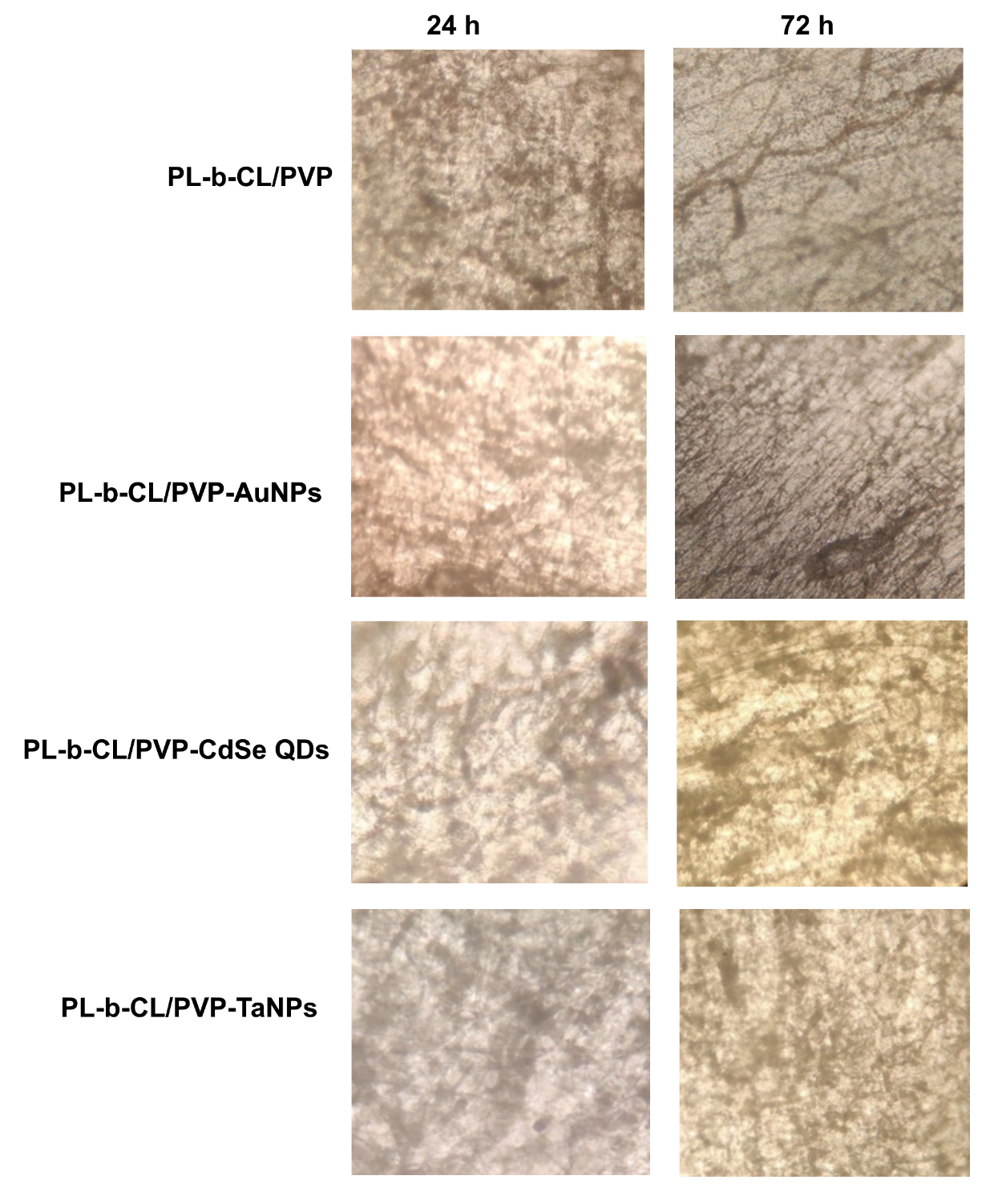


**Figure S11. The morphology of different nanofibers in medium after 24 and 72 h.**
